# Identification and Accumulation of Phenolic Compounds in the Leaves and Bark of *Salix alba* (L.) and Their Biological Potential

**DOI:** 10.3390/biom10101391

**Published:** 2020-09-29

**Authors:** Ewelina Piątczak, Monika Dybowska, Elżbieta Płuciennik, Katarzyna Kośla, Joanna Kolniak-Ostek, Urszula Kalinowska-Lis

**Affiliations:** 1 Department of Biology and Pharmaceutical Botany, Faculty of Pharmacy, Medical University of Lodz, Muszyńskiego 1, 90-151 Łódź, Poland; ewelina.piatczak@umed.lodz.pl; 2 Department of Cosmetic Raw Materials Chemistry, Faculty of Pharmacy, Medical University of Lodz, Muszyńskiego 1, 90-151 Łódź, Poland; monika.dybowska@umed.lodz.pl; 3 Department of Molecular Carcinogenesis, Medical University of Lodz, Żeligowskiego 7/9, 90-752 Łódź, Poland; elzbieta.pluciennik@umed.lodz.pl (E.P.); katarzyna.kosla@umed.lodz.pl (K.K.); 4 Department of Fruit, Vegetable and Plant Nutraceuticals Technology, Wroclaw University of Environmental and Life Sciences, Chełmońskiego 37, 51-630 Wrocław, Poland; joanna.kolniak-ostek@upwr.edu.pl

**Keywords:** polyphenolic compounds, antioxidant activity, cytotoxic activity, fibroblast cell line, keratinocyte cell line

## Abstract

The study examines the phenolic compounds in hydromethanolic extracts of *Salix alba* (L.) leaves and bark as well as their antioxidant activity and cytotoxic potential. UPLC-PDA-Q/TOF-MS analysis showed a total of 29 phenolic compounds in leaves and 34 in bark. Total phenolic compound content was 5575.96 mg/100 g of dry weight (DW) in leaves and 2330.31 mg/100 g DW in bark. The compounds were identified as derivatives of phenolic acids (seven in leaves and five in bark), flavanols and procyanidins (eight in leaves and 26 in bark) and flavonols (14 in leaves and three in bark). Both extracts exhibited strong antioxidant potential, assessed by radical scavenging activity against 1,1-diphenyl-2-picrylhydrazyl (DPPH) and 2,2′-azinobis (3-ethylbenzothiazoline-6-sulfonic acid (ABTS), but the bark extract was even stronger than the ascorbic acid used as a standard. The cytotoxicity of both extracts was evaluated against human skin fibroblasts and human epidermal keratinocytes cell lines using the Presto Blue cell viability assay. The keratinocytes were more resistant to tested extracts than fibroblasts. The leaf and bark extracts at concentrations which exhibited antioxidant activity were also not toxic against the keratinocyte cell line. Thus, *S. alba* extracts, especially the leaf extract, offer promise as a nontoxic natural antioxidant, in cosmetic products or herbal medicines, and as a source of bioactive secondary metabolites.

## 1. Introduction

The last few years have seen a growth in the need for natural drugs obtained from medicinal plants as an alternative to synthetic products [1]. A number of these bioactive compounds serve as starting points for the development of new drugs [2]. Clinical studies have shown that some herbal medicines are better tolerated by patients and show fewer side effects than synthetic derivatives [3].

Willow bark extract has been used for thousands of years as an analgesic, antipyretic and anti-inflammatory agent. Records suggest that, as long as 6000 years ago, white willow was used in Mesopotamia. Hippocrates recommended chewing willow bark for patients suffering from fever, inflammation and pain [4]. Currently, willow bark is used to treat many different types of pain, such as rheumatic pain, back pain, toothache, as well as menstrual cramps. It is also used to relieve fever, sore throat, headache and flu, mainly due to the presence of salicin, which is a natural nonselective COX-1 and COX-2 inhibitor [5].

Although willow bark extracts are generally standardized to salicin, other compounds in the extracts, including polyphenols, may also play prominent roles in areas such as cosmetology [6,7]. Recently, barks of different vascular woody plants are considered as a valuable source of bioactive compounds, exhibiting wide biological activities, including, among others, antioxidant, anti-inflammatory, and antitumor properties. Many studies confirm that the wide spectrum of bioactivities of bark extracts is connected mainly with the presence of (poly)phenolic compounds [8,9]. Polyphenolic compounds demonstrate antimutagenic, anticarcinogenic, and antiaging properties; they also demonstrate very strong antioxidant properties by capturing reactive oxygen species [10] and can hence protect biological systems against the oxidative stress that can cause cancer, atherosclerosis, diabetes, accelerated aging and Alzheimer’s disease, among others [11,12].

White willow (*Salix alba* L.)—Salicaceae is a small tree that naturally occurs in Europe, Asia and North Africa. The species is common in cold and low-temperature regions of the northern hemisphere, and in Poland, where it mainly grows on riverbanks on moist soils [13].

The three species: *Salix purpurea*, *S. daphnoides* and *S. fragilis* are the main sources of the drug willow bark—*Salicis Cortex* and are widely described in the literature [14]. Reports on the polyphenolic compounds present in other *Salix* species are incomplete and limited. Only a few compounds have been reported so far in the bark and/or leaves of other than *S. alba* willow species, e.g., *S. purpurea*, *S. daphnoides*, *S. phylicifolia*, *S. myrsinites*, *S. glauca*, *S. fragilis*, *S. lapponum*, *S. borealis* and *S. reticulata* [15,16,17,18,19]. Data on the chemical composition and biological activity of *S. alba* are limited [6,20,21]. Karl et al. [20] detected several flavonoid derivatives in *S. alba.* Zaiter et al. [21] quantified the main compounds (lipids, protein, ashes, carbohydrates) from hydromethanolic extracts of *S. alba* bark powders obtained after grinding and sieving. Sulaiman et al. [6] determined the total phenolic content in ethanolic extract from *S. alba* bark by the Folin–Ciocalteu method, and assayed various biological activities, including radical scavenging activity by DPPH assay.

However, the phytochemical profile of white willow leaf extract has not been investigated so far. Therefore, the aim of the present study is to perform a qualitative and quantitative analysis of the polyphenolic profile of leaf and bark extracts of *S. alba* using UPLC-PDA-Q/TOF-MS. The paper also compares the biological potentials of the two analyzed extracts. It examines their radical scavenging activity using 1,1-diphenyl-2-picrylhydrazyl (DPPH) and 2,2′-azinobis(3-ethylbenzothiazoline-6-sulfonic acid (ABTS) assays. In addition, in this study, we also investigated, for the first time, the cytotoxic activity of the two extracts against two lines of human skin cell lines (human skin fibroblast and human epidermal keratinocytes) in Presto Blue cell viability assay.

## 2. Materials and Methods

### 2.1. Reagents and Standards

TPTZ (2,4,6-tris(2-pyridyl)-s-triazine), ABTS (2,2′azino-bis(3-ethylbenzothiazoline-6-sulphonic acid), potassium persulfate, DPPH (2,2-diphenyl-1-picrylhydrazyl), phosphate buffer solution at pH 7.4, FeCl_3,_ acetate buffer at pH 3.6, methanol, were supplied by Sigma-Aldrich (Merck, Darmstadt, Germany). HCl, ascorbic acid and chloroform were supplied by POCH (Avantor, Gliwice, Poland).

Formic acid, methanol, 1-caffeoylquinic, 3-caffeoylquinic and 5-caffeoylquinic acid were purchased from Sigma-Aldrich (Steinheim, Germany). Acetonitrile was purchased from Merck (Darmstadt, Germany). Isorhamnetin 3-*O*-rutinoside, isorhamnetin 3-*O*-galactoside, quercetin 3-*O*-galactoside, quercetin 3-*O*-glucoside, quercetin 3-*O*-rutinoside, (+)-catechin and procyanidin B2 were purchased from Extrasynthese (Lyon, France).

### 2.2. Plant Material

Bark and leaves of *Salix alba* (L.) were collected in Lębork, Poland (54°32′ N 17°45″ E) (22 April 2019). The plant was identified by E. Piątczak according to Rutkowski [22]. The voucher specimen was deposited at the Department of Biology and Pharmaceutical Botany, Medical University of Łódź, Poland (No. EP/S.a./2019). The plant material was air dried at room temperature for two weeks protected from light.

### 2.3. Preparation of Extracts

Dry plant material (1 g was used for antioxidant experiments and 100 mg was weighted for UPLC analysis) was pre-extracted with chloroform overnight to remove chlorophyll. After filtration, the remaining plant material was extracted three times with a 30 mL methanol/water mixture 7:3 (*v*/*v*) for 30 min in an ultrasonic bath at 40 °C. Then, the mixture was filtered through a paper filter, the extracts were combined and evaporated to dryness under reduced pressure. The yields of the extracts were as follows: 32.8% (leaf extract) and 34.6% (bark extract).

### 2.4. Phytochemical Profiling

#### UPLC-PDA-Q/TOF-MS Analysis of Polyphenols

The polyphenols in the *S. alba* extracts were identified using an ACQUITY Ultra Performance LC system equipped with a photodiode array detector with a binary solvent manager (Waters Corporation, Milford, MA, USA) with a mass detector G2 Q-TOF micro mass spectrometer (Waters, Manchester, UK) equipped with an electrospray ionization (ESI) source operating in negative mode. Individual polyphenols were separated using a UPLC BEH C18 column (1.7 mm, 2.1 × 100 mm, Waters) at 30 °C. A 10 µL volume of samples were injected, and the elution was completed in 15 min with a sequence of linear gradients and constant flow rates of 0.42 mL/min. The mobile phase consisted of solvent A (0.1% formic acid, *v*/*v*) and solvent B (100% acetonitrile). The linear gradient was as follows: 0.0–1.0 min, 99% A, 0.42 mL/min (isocratic), 1.0–12.0 min, 65.0% A, 0.42 mL/min (linear), 12.0–12.5 min, 99% A, 0.42 mL/min (linear), 12.5–13.5 min, 99% A, 0.42 mL/min (isocratic). The analysis was carried out using full scan, data-dependent MS scanning from *m*/*z* 100–1500. Leucine-enkephalin was used as the reference compound at a concentration of 500 pg/mL, and the [M-H]^−^ ion at 554.2615 Da was detected. The [M-H]^−^ ions were detected during a 15 min analysis performed within ESI–MS accurate mass experiments, which were permanently introduced via the LockSpray channel using a Hamilton pump. The lock mass correction was 1000 for the mass window. The mass spectrometer was operated in negative-ion mode, set to the base peak intensity (BPI) chromatograms, and scaled to 12,400 counts per second (cps) (100%). The optimized MS conditions were as follows: capillary voltage of 2500 V, cone voltage of 30 V, source temperature of 100 °C, desolvation temperature of 400 °C and desolvation gas (nitrogen) flow rate of 600 L/h.

Collision-induced fragmentation experiments were performed using argon as the collision gas, with voltage ramping cycles from 0.3 to 2 V. Characterization of the single components was carried out based on the retention time and the accurate molecular masses. Each compound was optimized to its estimated molecular mass in the negative mode, before and after fragmentation. The data obtained from UPLC–MS were subsequently entered into the MassLynx 4.0 ChromaLynx Application Manager software (Waters).

The runs were monitored at the following wavelengths: flavan-3-ols and procyanidins at 280 nm, phenolic acids at 320 nm and flavonols at 360 nm. The PDA spectra were measured over the wavelength range of 200–600 nm in steps of 2 nm. The retention times and spectra were compared to those of the authentic standards.

The quantification of phenolic compounds was performed by external calibration curves, using reference compounds selected based on the principle of structure-related target analyte/standard (chemical structure or functional group). The calibration curve for 3-caffeoylquinic acid was used to quantify caffeoylquinic acid derivatives. The calibration curve for caffeic acid was used to quantify caffeoylhexose derivatives. Then, 1-caffeoylquinic and 5-caffeoylquinic acids were quantified with their own standards. The calibration curve for procyanidin B2 was used to quantify all B-type procyanidins. (+)-catechin and (−)-epicatechin were quantified with (+)-catechin standard. The calibration curves of quercetin rutinoside, 3-*O*-glucoside and 3-*O*-galactoside were used to quantify quercetin derivatives. For isorhamnetin quantification, isorhamnetin 3-*O*-rutinoside and 3-*O*-glucoside were used.

All determinations were done in triplicate (*n* = 3).

### 2.5. Antioxidant Activity

#### 2.5.1. ABTS Assay

The antioxidant activity was determined using the ABTS radical cation decolorization test according to Re et al. [23] and Guss et al. [24] using a Shimadzu UV-1800 UV/Vis spectrophotometer. An ABTS stock solution was prepared by 19.5 mg ABTS (Sigma-Aldrich Sp. z o.o., Poznań, Poland) mixed with 50 mM phosphate buffer at pH 7.4 and 3.3 mg potassium persulfate (Sigma-Aldrich Sp. z o.o., Poznań, Poland). This mixture was kept in the dark at room temperature for 14 h before use. The ABTS stock solution was diluted with phosphate buffer at pH 7.4 to give an absorbance of the negative control (0.9 ± 0.05) at 734 nm. The assay was performed by adding 0.5 mL of each willow extract (from leaves and bark, separately) at concentrations of 7.5, 10.0, 15.0, 25.0 and 50.0 µg/mL to 0.5 mL of ABTS solution. The absorbance was measured after 10 min at 734 nm. The results were expressed as EC_50_, the concentration of the sample (in µg of dry extract/mL) at which 50% of maximum scavenging activity was recorded. Ascorbic acid was used as an antioxidant standard.

#### 2.5.2. DPPH Assay

Radical scavenging activity of samples was evaluated by spectrophotometry by monitoring the decrease of absorbance at 517 nm of methanolic solution of the radical DPPH (2,2-diphenyl-1-picrylhydrazyl) incubated for 30 min in the darkness in the absence or in the presence of plant extracts [25,26]. The DPPH solution was diluted with methanol/water mixture 7:3 (*v*/*v*) to give an absorbance of the negative control (0.9 ± 0.05) at 517 nm. The assays were performed by adding 0.5 mL of each plant extract at concentrations of 1.0, 5.0, 10.0, 15.0, 20.0, 25.0, 50.0, 100.0 (µg/mL) to 0.5 mL of DPPH solution. The concentration of the extract (µg of dry extract/mL), necessary to reduce the initial concentration of DPPH by 50% (EC_50_) under the experimental conditions was calculated. Ascorbic acid was used as a positive control.

### 2.6. Cytotoxic Activity

#### 2.6.1. Cell Lines and Culture Medium

Cytotoxicity was measured against two human cell lines: 1BR.3.N (human skin fibroblasts, ECACC, Co. 90020508) and human epidermal keratinocytes, neonatal cells (Millipore, co. SCCE020).

The human skin fibroblast (1BR.3.N) cells were maintained in EMEM (EBSS) culture medium supplemented with 2 mM glutamine, 1% nonessential amino acids (NEAA), 10% fetal bovine serum (FBS), 1% antibiotic–antimycotic.

Human epidermal keratinocytes were cultured in EpiGRO™ human epidermal keratinocyte complete medium with 6 mM of L-glutamine, 0.40% EpiFactor P, 1.0 μM epinephrine, 0.5 ng/mL of TGF-α, 100 ng/mL hydrocortisone hemisuccinate, 5 μg/mL of insulin and apo-transferrin.

The cells were cultured at 37 °C in a humidified 5% CO_2_ incubator.

#### 2.6.2. Assay for Cytotoxic Activity

The extract of *S. alba* leaf and bark were tested for in vitro cytotoxicity against 1BR.3.N and human epidermal keratinocytes, Neonatal cells by Presto Blue cell viability assay [27] according to the manufacturer’s instructions.

The stock solution (1 mg/mL) of plant extracts were dissolved in a mixture of methanol/water (7:3 *v*/*v*). The final dilution of the stock solutions was made in culture medium. Cells were also treated with vehicle control (i.e., equivalent amounts of a 7:3 methanol/water solution mixture without extracts).

Keratinocytes and fibroblasts were seeded into 96-well plates in the full medium at a density of 10^4^ cells/well. Twenty-four hours after seeding, the cells were treated with different concentrations of plant extracts (from 5 to 200 μg/mL) or with vehicle control in either full medium (for keratinocytes) or medium without FBS (for fibroblasts). After 24 h of incubation, cytotoxicity was evaluated using the Presto Blue cell viability assay on a VICTOR™ ×4 Multilabel Plate Reader (Perkin Elmer, London, UK). IC_50_ values, which means the extract concentration exhibited 50% cell growth inhibition, were calculated using the GraphPad Prism 5.01 software tool.

### 2.7. Statistical Analysis

Each experiment was performed in triplicate and repeated at least three times. All data are presented as means ± standard deviation (SD). EC_50_ values (antioxidant activity) were calculated using Microsoft Excel software. IC_50_ values (cytotoxic activity) were calculated using the GraphPad Prism 5.01 software tool.

The statistical significance between both extracts was determined using a nonparametric, distribution-free Mann–Whitney U-test or the Kruskal–Wallis test. Differences between means were considered significant at *p* ≤ 0.05 for both tests. All the statistical analyses were calculated using Statistica software (version 13.1, 2016, STATSoft, Cracow, Poland).

## 3. Results and Discussion

### 3.1. Phytochemical Profiling

#### 3.1.1. Identification of Phenolics in *Salix alba* Extracts

**Figure 1:**
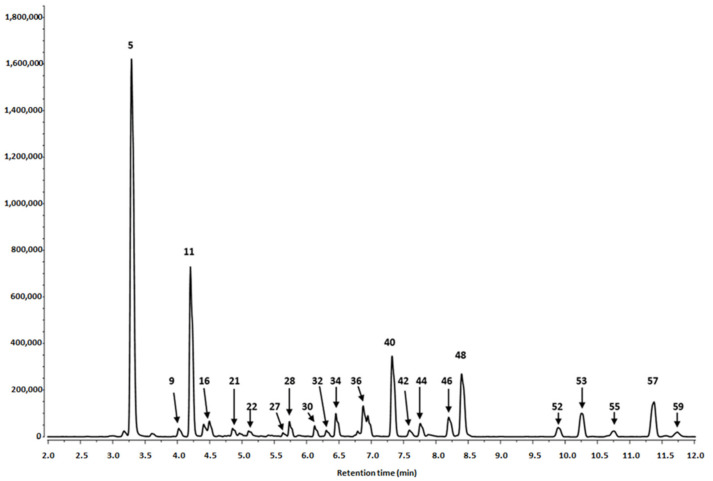
Segment from 2.00 to 12.00 min of LC-DAD chromatogram at 340 nm of *S. alba* leaves. Peak number identities are displayed in Table 1.

**Figure 2:**
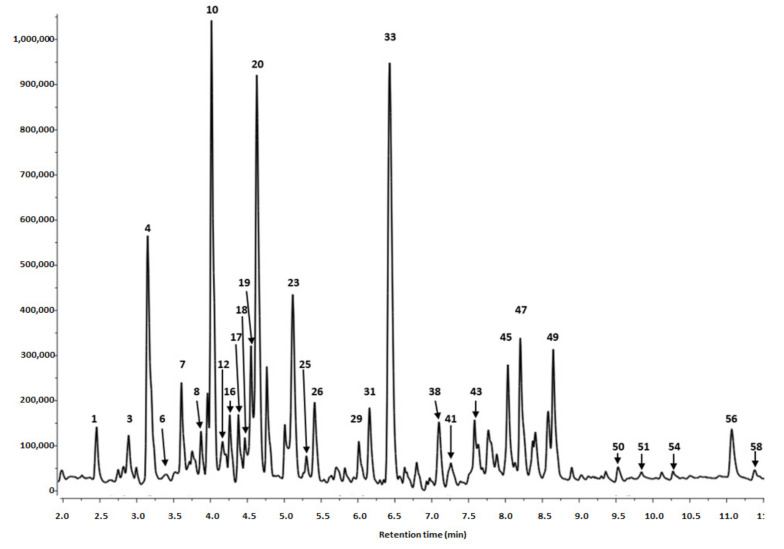
Segment from 2.00 to 11.50 min of LC-DAD chromatogram at 280 nm of S. alba bark. Peak number identities are displayed in Table 1.

**Figure 3:**
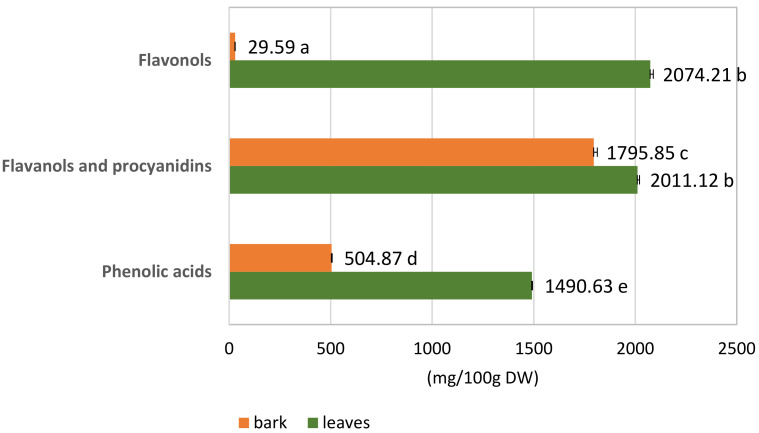
Contents (mg/100 g DW) ± SD of phenolic compounds in hydromethanolic extracts of leaves and bark of *S. alba.* Means with the same letter are not statistically different at *p* ≤ 0.05 in the Kruskal–Wallis test.

In the present study, the phytochemical profile of hydromethanolic extracts of *S. alba* leaves and bark was analyzed using UPLC-PDA-Q/TOF-MS. The results of the qualitative and quantitative analyses are summarized in Table 1 and Figure 1, Figure 2 and Figure 3.

Twenty-nine phenolic compounds were identified in the leaf extracts and 35 in the bark extracts. They included derivatives of phenolic acids, flavanols, procyanidins and flavonols.

##### Phenolic Acid Derivatives

Seven derivatives of caffeic acid (peaks **1**, **5, 9, 11, 15, 21** and **24**) were detected in *S. alba* leaf extract and five (peaks **1–3**, **11** and **13**) in the bark extract. Among them, caffeic acid hexoses, caffeoyl hexose-deoxyhexosides, caffeoylquinic acid isomers and dimers were identified. Three isomeric compounds found in leaves, *viz.* 1-*O*-caffeoylquinic acid, 3-*O*-caffeoylquinic acid (neochlorogenic acid) and 5-*O*-caffeoylquinic acid (chlorogenic acid), peaks **9**, **11** and **24** (t_r_ = 4.04, 4.12 and 5.14 min), were identified by comparison with authentic standards (Table 1). They exhibited a pseudomolecular [M-H]^−^ ions at *m*/*z* 353 and fragmentation ions at *m*/*z* 191 and *m*/*z* 179, corresponding to quinic acid and caffeic acid residues, respectively. One of the mentioned derivatives, i.e., 3-*O*-caffeoylquinic acid, peak **11** (t_r_ = 4.12), was also present in the bark.

The further derivatives, two isomeric caffeoylquinic acid dimers, were identified only in leaves. Compounds **15** and **21** (t_r_ = 4.24 and 4.77 min) were represented by a pseudomolecular [M-H]^−^ ions at *m*/*z* 707. The presence of fragmentation ions at *m*/*z* 353 due to the loss of caffeoylquinic acids (−353 u) confirmed their dimeric structure.

Caffeoylquinic acid derivatives (1- and/or 3-, 4-, 5-caffeoylquinnic acids) have previously been detected in bud extracts of *Salix pyrolifolia* [28] and in the leaves of other species, for example *Morus alba, Morus rubra* [29], *Salvia verticillata* [30], *Gaultheria procumbens* [31], *Sorbus domestica* [32].

Caffeic acid hexoses were detected in both the bark and leaves. Compounds **1**, **2** and **13** (t_r_ = 2.51, 2.72 and 4.17 min) found in the bark and the compound **1** (t_r_ = 2.51 min) detected in leaves had main pseudomolecular ions [M-H]^−^ at *m*/*z* 341 and the fragmentation ions at *m*/*z* 179 which correspond to the loss of hexose residues (162 u).

Compounds **3** (t_r_ = 2.76 min) and **5** (t_r_ = 3.26 min), identified as a caffeoyl hexose-deoxyhexosides, were detected in both extracts, bark and leaves, respectively. They exhibited pseudomolecular ions [M-H]^−^ at *m*/*z* 487. The fragmentation pattern showed ions at *m*/*z* 308 [M-H-179]^−^ and at *m*/*z* 179 [M-H-162-146]. The first was obtained by the loss of the caffeic acid aglicone (179 u). The residual part of the compound was composed of a hexose residue (162 u) and a deoxyhexoside residue (146 u).

##### Flavanols and Procyanidins

Twenty-six compounds from the flavanols and procyanidins group were found in the *S. alba* bark extract. These compounds consisted mainly (epi)catechin derivatives such as A-type and B-type procyanidins (dimers, trimers and tetramers) and their digallates; (epi)catechin-(epi)gallocatechins or (epi)catechin methyl-hexosides. Much fewer compounds from this group of derivatives were found in leaves that included two epigallocatechins, A-type procyanidin dimer and five B-type procyanidins (one dimer, three trimers and one pentamer).

Free (+)-catechin and (−)-epicatechin were present only in the bark extract. Peaks **19** and **20** (t_r_ = 4.55 and 4.74 min), were detected with pesudomolecular ions [M-H]^−^ at *m*/*z* 289. Two epigallocatechins, peaks **22** (t_r_ = 5.08) and **28** (tr = 5.75), contained in leaves had the main ion [M-H]^−^ at *m*/*z* 305. Three isomers of (epi)catechin-(epi)gallocatechins, i.e., peaks **4**, **6** and **7** (t_r_ = 3.12, 3.45 and 3.69 min) were present only in the bark; they were identified by pseudomolecular ions [M-H]^−^ at *m*/*z* 593 and fragmentation ions at *m*/*z* 289, attributed to (epi)catechin. (+)-Catechin and (−)-epicatechin are found in a range of fruits, beans and chocolate [33]. The compounds have been also widely distributed in leaf extracts of several *Salix* species [15], including *S. alba*, *S. subserrata*, *S. fragilis*, *S. daphnoides*, *S. caprea*, *S. cinerea* [19,34]. Catechin was also a predominant compound in water extracts of *S. aegyptiaca* leaves [35] and buds of *S. pyrolifolia* [28].

B-type procyanidins (dimer, trimers, tetramers and pentamer) were detected in both the bark and leaves. The B-type procyanidin dimer (peak **36** of t_r_ = 6.79), trimers (peaks **16, 26, 27** and **30** of t_r_ = 4.30, 5.45, 5.64 and 6.10 min), tetramers (peaks **29** and **31** of tr = 6.07 and 6.20 min) and pentamer (peak **34** of t_r_ = 6.47 min) from leaves and bark showed pseudomolecular ions at *m*/*z* 575 (dimer), *m*/*z* 865 (trimers), *m*/*z* 1153 (tetramers) and *m*/*z* 1441 (pentamer), respectively. Each of these five B-type procyanidins was represented by fragmentation ion at *m*/*z* 287 typical for the monomer of catechin and its multiple.

In the bark and leaves a multitude of isomeric A-type procyanidins dimers (peaks **10**, **12**, **14**, **18**, **33** and **45** of t_r_ = 4.09, 4.16, 4.20, 4.48, 6.45 and 8.08 min, respectively) showed ions at *m*/*z* 577 and 289; trimers (peaks **17**, **25** and **47** of t_r_ = 4.39, 5.30 and 8.17 min) at *m*/*z* 865, 577 and 289 and tetramer (peak **43** of t_r_ = 7.60 min) at *m*/*z* 1152, 863, 577 and 289. All these mentioned values of peaks were a multiplication factor of the mass of the monomeric (epi)catechin equals to 287.

Proanthocyanidins are oligomers or polymers of flavan-3-ols, which are commonly distributed in plants, for example in fruits (apple, grape, cranberry), red wine, cinnamon and the hawthorn inflorescences [36]. The metabolites exhibit various biological activities, including antihypertensive, antioxidative, and anti-inflammatory properties [15]. The possible mechanism of anti-inflammatory action of proanthocyanidin dimers, B1 and B2, may be connected with the inhibition of transcription of nuclear factor-kappa B (NF-κB) [37]. The B-type procyanidin identified in white willow leaves in the present study were proanthocyanidins consisting of catechin and/or epicatechin units. Earlier, the presence of procyanidins (oligomers and polymers) (B and C-type) has been confirmed in the leaves of three willow species, including *S. alba* bark extracts [19,21,38]. Among other species, several procyanidin A and B-type derivatives have been identified in *Gaultheria procumbens* leaf extracts [39] and six different dimers and three different trimers of B-type procyanidin have been identified in *Sorbus demestica* leaf extract [32].

Peaks **38**, **41**, **49** and **50** (t_r_ = 7.14, 7.28, 8.68 and 9.56 min) detected only in the bark, were identified as (epi)catechin methyl-hexosides. They showed fragmentation ions at *m*/*z* 289, corresponding to (epi)catechin aglycon resulting from a loss of methyl-hexoside [M-H-176]^−^ moiety from a pseudomolecular ion at *m*/*z* 465 [M-H]^−^.

A-type procyanidin dimer digallate (peak **8**, t_r_ = 3.87 min) and procyanidin trimer digallate (peak **23**, t_r_ = 5.12 min) demonstrated pseudomolecular ions at *m*/*z* 881 and 1169, respectively. Each compound demonstrated fragmentation ions at *m*/*z* 289 assigned to epichatechin. The fragmentation patterns of trimeric compound (peak **23**) at *m*/*z* 289, 577 and 865 confirmed the presence of monomeric, dimeric and trimeric epicatechin, respectively, and the loss of digallate residue (304 u) from a main pseudomolecular ion at *m*/*z* 1169.

The last compound present in the willow bark—(epi)catechin-ethyl trimer (peak **56**, t_r_ = 11.09 min) with the main ion at *m*/*z* 893 and fragmentation ions at *m*/*z* 603 and 289 revealed that the compound fragmentized into a (epi)catechin-ethyl (317 u) moiety and two (epi)catechins (289 u).

##### Flavonols

The *S. alba* leaf extract was particularly rich in flavonol derivatives, while the bark extract had a negligible amount of the compounds, including only three derivatives.

Fourteen flavonols were detected in the leaf extracts: eight quercetin derivatives (peaks **32**, **35**, **37**, **39**, **42**, **44**, **46** and **48**) and six isorhamnetin derivatives (peaks **40**, **52, 53, 55, 57** and **59**) (Table 1). None of them were contained in the bark extract.

Quercetin derivatives were represented by quercetin 3-*O*-rutinoside (rutin), -3-*O*-galactoside, -3-*O*-glucoside, methyl-pentoside and -acylated-hexosides. Each of the derivatives demonstrated the typical quercetin fragment at *m*/*z* 301. Peak **32** (t_r_ = 6.39 min) was identified as quercetin 3-*O*-rutinoside (rutin). The rutin exhibited a pseudomolecular ion [M-H]^−^ at *m*/*z* 609 and a fragmentation ion at *m*/*z* 301 obtained by the loss of a rutinoside residue [M-H-308]^−^. The compound is commonly present in plants from various taxa, for example in aerial parts of *Salvia verticillata* [30], *Gaultheria procumbens* [31] and *Morus alba* [29] as well as in the leaves of white willow [20].

Peaks **42** and **44** (t_r_ = 7.54 and 7.70 min), assigned to quercetin -3-*O*-galactoside and quercetin -3-*O*-glucoside had main ions at *m*/*z* 463 and fragmentation ions attributed to the loss of a hexoside moiety (−162 u) at *m*/*z* 301 [M-H-hexoside]^−^. Both the mentioned glycosides of quercetin have been earlier detected in leaf extracts from six different northern willow species [18] as well as in buds of *Salix pyrolifolia* [28]. Quercetin -3-*O*-glucoside, together with another quercetin derivative, quercetin7′,3-dimethylether 3-*O*-glucoside, has been earlier detected in the leaves of *S. alba* [20]. Quercetin -3-*O*-glucoside, together with several other quercetin and kaempferol glycosides have been also identified in the leaves of *Morus alba*, *M. rubra*, *Gaultheria procumbens* [29,31].

Other quercetin derivatives, four isomeric acylated-hexosides, peaks **37**, **39**, **46**, **48** (t_r_ = 7.13, 7.18, 8.14, 8.43 min), and methyl-pentoside, peak **35** (t_r_ = 6.62 min), had the typical quercetin ion fragment at *m*/*z* 301 and main ions [M-H]^−^ at *m*/*z* 651, 651, 505, 505 and 447, respectively. Two quercetin-acylated-deoxyhexosides (peaks **37** and **39**) of the main ions at *m*/*z* 651 gave fragmentation ions at *m*/*z* 446 and 301 suggesting that the compounds initially lost some hexoside residue together with an acetyl group (−162 u and −42 u) and subsequently a deoxyhexoside residue, e.g., rhamnose moiety (−146 u). Two quercetin-acylated-hexosides (peaks **46** and **48**) had pseudomolecular ions at *m*/*z* 505 and fragmentation ions at *m*/*z* 301 and one of them additionally had an ion at *m*/*z* 463, which revealed both compounds lost acetyl group (−42 u) and some hexoside moiety (−162 u). The last quercetin derivative, methyl-pentoside (peak **35**), gave ions at *m*/*z* 447 and at 301, which confirmed the loss of methyl-pentoside residue (−146 u).

The isorhamnetin derivatives included -3-*O*-rutinoside, peak **40** (t_r_ = 7.22 min); -3-*O*-galactoside, peak **53** (t_r_ = 10.20 min); and –acylated-hexosides, peaks **52**, **55**, **57**, **59** (t_r_ = 9.80, 10.63, 11.24, 11.63 min), that had a [M-H]^−^ *m*/*z* at 623, 477 and 519 [477 + 42 (acetyl group –C(O)CH_3_)], respectively. All of them possess the typical isorhamnetin (or deprotonated isorhamnetin) fragment at *m*/*z* 315 (or at *m*/*z* 314) formed by the cleavage of hexoside residues, i.e., rutinoside (−308 u), -galactoside (−162 u) and –acylated-hexosides (−204 u [−162 u and −42 u]), from the isorhamnetin glycosides. Additionally, the -acylated-hexoside derivatives possess a fragment at *m*/*z* 299 corresponding to demethylated isorhamnetin. Isorhamnetin derivatives were often present in the leaves of various species of willows. Isorhamnetin-3-*O*-glucoside and isorhamnetin-3-*O*-rutinoside have been earlier detected in the leaves of *S. alba* [20]. Isorhamnetin-3-*O*-glucoside has been earlier found in extracts of leaves of six *Salix* species grown in Finnish Lapland [18] and in the leaves of *S. viminalis* [40]. A different derivative of isorhamnetin (isorhamnetin-3-*O*-rhamnoside) has been also identified in buds of *S. pyrolifolia* [28].

As for the content of flavonols in the bark extract, only three derivatives were detected, i.e., quercetin 3-*O*-hexoside, kaempferol pentoside and kaempferol 3-*O*-galactoside.

Quercetin 3-*O*-hexoside, peak **51** (t_r_ = 9.69 min), had a typical quercetin ion fragment at *m*/*z* 301 obtained by a cleavage of a hexoside moiety (162 u) from the main ion [M-H]^−^ at *m*/*z* 463.

Two kaempferol derivatives, peaks **54** and **58** (t_r_ = 10.27 and 11.33 min), displayed pseudomolecular ions [M-H]^−^ at *m*/*z* 423 and 447, respectively. The fragmentation ions at *m*/*z* 287 confirmed the presence of a characteristic protonated kaempferol in the mentioned derivatives and the cleavage of a pentoside (−132 u) and galactoside (−162 u) moieties, respectively, from the main ions.

#### 3.1.2. Quantitative Analysis of the *S. alba* Leaf and Bark Extracts

Quantitative analysis of dry extracts of *S. alba* leaves and bark was conducted for the presence of polyphenolic compounds, such as phenolic acids, flavanols and procyanidins, and flavonols (Table 1; Figure 3). The analyses were performed based on external calibration curves using selected reference compounds (Materials and Methods: Section 2.4). The concentration of the individual substances was expressed in mg/100 g DW.

The two extracts had different phenolic compound contents. *S. alba* leaf extract was a richer source of total active phenolic ingredients than bark extract: 5575.96 mg/100 g DW in the leaves compared to 2330.31 mg/100 g DW in the bark, i.e., 58.2% higher content.

Flavonol content was up to 98.5% higher in the leaves than the bark, with only trace amounts in the bark. Similarly, the leaves also had 66% more phenolic acids than the bark. Total flavanols and procyanidins content was also 11% higher in the leaves than in the bark.

In the leaves, the number of flavonols was 2074.21 mg/100 g DW, similarly as the content of the flavanols and procyanidins, which was equal to 2011.12 mg/100 g DW. Among the flavonols, quercetin derivatives accounted for 56.3% (1168.33 mg/100 g DW); the remaining 43.7% comprised isorhamnetin derivatives (905.88 mg/100 g DW). Phenolic acids constituted a significant amount of this extract and were equal to 1490.63 mg/100 g DW.

In the bark, the flavanols and procyanidins constituted as much as 77.1% of all considered phenolic compounds (1795.85 mg/100 g DW). Phenolic acid content was about 3.5 fold lower (504.87 mg/100 g DW) than in leaves. The content of flavonols in the bark was negligible (29.59 mg/100 g DW) (Table 1).

### 3.2. The Antioxidant Activity

The antioxidant activity of the hydromethanolic leaf and bark extracts was determined by DPPH and ABTS^−^ radical scavenging assays (Table 2).

The bark extract of *S. alba* exhibited stronger antioxidant activity (48% in DPPH and 33% in ABTS), than the leaf extract. However, leaf extract activity was also very strong, and similar (at *p* ≤ 0.05) to the natural reference antioxidant: ascorbic acid (EC_50_ = 28 μg/mL in DPPH assay and 65 μg/mL in ABTS assay) (Table 2). The bark extract demonstrated lower EC_50_ values (EC_50_ = 13.5 μg/mL in DPPH assay and 21.5 μg/mL in ABTS assay) than the reference standard, indicating even stronger antioxidant activity than ascorbic acid. A previous DPPH measurement of antioxidant activity in *S. alba* bark extract showed slightly lower activity (EC_50_ = 19.1 μg/mL) [41]. Another study reported even lower antioxidant activity in DPPH (58–65 μg/mL) and ABTS (47–53 μg/mL) assays, depending on the granulometry classes of the powdered bark extract [21]. Among other *Salix* species, *S. atrocinerea* and *S. viminalis* bark extracts exhibited similar activities in the DPPH test (EC_50_ = 10.98 and 14.06 μg/mL, respectively) as in the present study. On the other hand, the bark extract of *S. fragilis* demonstrated lower activity (EC_50_ = 23.62 μg/mL) [42]. The results of the ABTS assay are comparable with those achieved earlier for other willow species, including *S. purpurea* barks (EC_50_ = 20 μg/mL) [43].

Our paper is the first to describe the antioxidant potential of leaf extract of white willow. The extracts from leaves of other *Salix* species, for example *S. subserrata* and *S. aegyptiaca* [34,35], exhibited lower antioxidant activity in DPPH assay than leaf extract of *S. alba* in our study. The differences in the antioxidant capacity observed between even the same species, in the same assay, could be caused by differences in phytochemical profile, time of harvesting or even extraction method of the plant material.

The greatest influence on the antioxidant properties of the willow extracts was probably exerted by phenolic compounds, present in each extract. These are mainly phenolic acid derivatives and flavonols (quercetin and isorhamnetin derivatives) in leaves. In the bark extract, catechin, epicatechin and A and B-type procyanidin derivatives are probably responsible mainly for the properties.

### 3.3. Cytotoxic Activity

The hydromethanolic extracts of *S. alba* leaves and bark were tested for in vitro cytotoxicity, using two human skin cell lines: human skin fibroblasts (1BR.3.N) and human epidermal keratinocytes, neonatal cells. These two cell lines were chosen to check the cytotoxic effect of tested extracts (in the range of concentrations from 5 to 200 µg/mL) on skin cells and check the possibility of external use of the extracts, for example in cosmetology.

**Figure 4:**
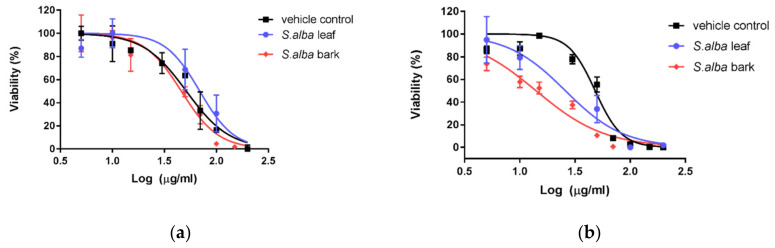
Effects of *S. alba* bark and leaf hydromethanolic extracts on keratinocyte (**a**) and fibroblast (**b**) cell line viability in Presto Blue cell viability assay.

The *S. alba* bark and leaf extracts demonstrated different cytotoxic activity against fibroblasts and keratinocytes (Figure 4a,b). The obtained IC_50_ values were higher for keratinocytes than for fibroblasts, which means that both extracts were less cytotoxic to keratinocytes than fibroblasts. The cytotoxic effect of both *S. alba* bark and leaves was similar as the vehicle control, i.e., equivalent amounts of methanol/water solution (7:3), for keratinocytes (IC_50_ values of 46.08 and 69.33 µg/mL, respectively, versus 51.51 µg/mL for vehicle control). In the case of fibroblasts, the cytotoxic activity of *S. alba* bark was almost twice that of leaves (IC_50_ values of 14.06 and 25.92 µg/mL, respectively, versus 48.22 µg/mL for vehicle control).

It can be concluded that keratinocytes were more resistant to the cytotoxic effect of both *S. alba* extracts (Figure 4a). However, in the case of fibroblasts, the leaf extract demonstrated lower cytotoxic potential than bark extract (Figure 4b).

However, it is important to note that both extracts exhibited significant antioxidant activity at concentrations that were not toxic to the keratinocytes (EC_50_ = 13.5 μg/mL in DPPH assay and 21.5 μg/mL in ABTS assay). Therefore, the extracts may be active ingredients of products used externally, which are safe for the epidermis. However, lower concentrations should be used in cosmetics acting deeper in the skin, because of the higher toxicity of the tested extracts, especially bark extract, to the fibroblast cells of the dermis.

In earlier studies, *S. alba* bark extract has demonstrated cytotoxic and genotoxic potential against different cell lines, for example the human leukemia (HL-60) cell line [6], human peripheral leukocyte cells and the human hepatoma cell line, with doses from 50 and 200 µg/mL [44]. However, other extracts from bark of *S. atrocinerea* and *S. fragilis* did not exert a cytotoxic effect (at 625 and 1250 µg/mL) against immortalized human keratinocytes (HaCaT) and mouse fibroblast (L929) cells [42]. The differences between cytotoxicity dose could be connected with differences in the solvent or cell line model. Our paper is the first investigation of the cytotoxic potential of *S. alba* extracts on skin cell lines.

## 4. Conclusions

Although extracts from *Salix* sp. are commonly used for treatment, mainly due to salicin content, the phytochemical profiles of polyphenol compounds in white willow leaves and bark extracts appear to have a potentially beneficial influence on human health due to the presence of quercetin glycosides, monomeric, dimeric and trimeric flavan-3-ol derivatives, including B-type procyanidins, as well as caffeoylquinic pseudodepsides. These specialized secondary plant metabolites are known to exhibit a wide range of biological activities. Moreover, both extracts of white willow described in the present study exhibited strong antioxidant activity at concentrations that were not toxic to the keratinocytes. Therefore, the extracts, especially leaf extract, may be used as a potential new source of bioactive polyphenols with possible applications in some antiaging cosmetics, which would be safe for the epidermis. Bark extracts, also rich in polyphenolic compounds, should be used at lower concentrations than leaf extracts, especially in cosmetics that penetrate deeper into the skin, because of their higher toxicity to fibroblasts. However, further studies are needed, particularly those connected with the potential for utilization and extract standardization.

## Figures and Tables

**Table 1:** Characteristic ions and phenolic compounds content in *Salix alba* hydromethanolic leaf and bark extracts (mg/100 g DW) by UPLC-PDA-Q/TOF-MS.

Peak No	Retention Time (t_r_)	λmax (nm)	[M-H]^−^ (*m*/*z*) ^a^	MS/MS Fragments (*m*/*z*) ^a^	Tentative Identification	Content (mg/100 g DW) ^c^ ± SD	
						Leaves	Bark
**Phenolic Acids**							
1	2.51	324	341	179	Caffeoylhexose I	2.98 ± 0.07	57.71 ± 0.09 *
2	2.72	324	341	179	Caffeoylhexose II	n.d.	24.71 ± 0.10 *
3	2.76	320	487	308/179	Caffeoyl hexose-deoxyhexoside I	n.d.	140.00 ± 0.22 *
5	3.26	320	487	308/179	Caffeoyl hexose-deoxyhexoside II	875.32 ± 1.18 *	n.d.
9	4.04	324	353	191/179	1-*O*-Caffeoylquinic acid ^b^	117.43 ± 0.88 *	n.d.
11	4.12	324	353	191/179	3-*O*-Caffeoylquinic acid ^b^	386.31 ± 1.26	41.96 ± 2.53 *
13	4.17	324	341	179	Caffeoylhexose III	n.d.	240.49 ± 0.46 *
15	4.24	325	707	353/191/179	Caffeoylquinic acid dimer I	60.63 ± 1.83 *	n.d.
21	4.77	325	707	353/191/179	Caffeoylquinic acid dimer II	10.11 ± 0.75 *	n.d.
24	5.14	324	353	191	5-*O*-Caffeoylquinic acid ^b^	37.85 ± 0.59 *	n.d.
						Sum 1490.63 ± 2.86	Sum 504.87 ± 2.58 *
**Flavanols and Procyanidins**							
4	3.12	280	593	407/289	(Epi)catechin-(Epi)gallocatechin I	n.d.	130.67 ± 1.16 *
6	3.45	280	593	407/289	(Epi)catechin-(Epi)gallocatechin II	n.d.	10.09 ± 0.19 *
7	3.69	280	593	407/289	(Epi)catechin-(Epi)gallocatechin III	n.d.	59.79 ± 5.79 *
8	3.87	279	881	407/289	A-type procyanidin dimer digallate	n.d.	48.66 ± 0.50 *
10	4.09	280	577	407/289	A-type procyanidin dimer ^b^ I	n.d.	224.33 ± 1.58 *
12	4.16	281	577	407/289	A-type procyanidin dimer ^b^ II	241.31 ± 1.03	22.33 ± 0.57 *
14	4.20	280	577	289	A-type procyanidin dimer ^b^ III	n.d.	9.22 ± 0.90 *
16	4.30	277	865	577/287	B-type procyanidin trimer I	241.43 ± 1.16	22.90 ± 0.46 *
17	4.39	277	865	577/289	A-type procyanidin trimer I	n.d.	25.87 ± 0.18 *
18	4.48	281	577	407/289	A-type procyanidin dimer ^b^ IV	n.d.	14.27 ± 5.63 *
19	4.55	280	289		(+)-Catechin	n.d	63.79 ± 2.60 *
20	4.74	280	289	245	(−)-Epicatechin ^b^	n.d.	176.49 ± 14.02 *
22	5.08	281	305	221/179	Epigallocatechin ^b^ I	319.52 ± 1.62 *	n.d.
23	5.12	279	1169	865/577/289	A-type procyanidin trimer digallate	n.d.	99.25 ± 0.04 *
25	5.30	277	865	577/289	A-type procyanidin trimer II	n.d.	15.07 ± 0.08 *
26	5.45	277	865	577/287	B-type procyanidin trimer II	n.d.	40.79 ± 1.01 *
27	5.64	280	865	577/287	B-type procyanidin trimer III	124.37 ± 9.44 *	n.d.
28	5.75	280	305	221/219/179	Epigallocatechin ^b^ II	324.69 ± 0.88 *	n.d.
29	6.07	281	1153	865/577/287	B-type procyanidin tetramer I	n.d.	25.54 ± 0.04 *
30	6.10	277	865	577/287	B-type procyanidin trimer IV	246.46 ± 1.69 *	n.d.
31	6.20	281	1154	577/287	B-type procyanidin tetramer II	n.d.	56.07 ± 1.51 *
33	6.45	280	577	289	A-type procyanidin dimer ^b^ V	n.d.	198.24 ± 0.06 *
34	6.47	281	1441	864/575/287	B-type procyanidin pentamer	118.11 ± 0.13 *	n.d.
36	6.79	279	575	287	B-type procyanidin dimer ^b^ I	395.23 ± 0.73 *	n.d.
38	7.14	280	465	289	(Epi)catechin methyl-hexoside I	n.d.	68.55 ± 1.12 *
41	7.28	280	465	289	(Epi)catechin methyl-hexoside II	n.d.	56.78 ± 0.99 *
43	7.60	280	1152	863/577/289	A-type procyanidin tetramer	n.d	33.45 ± 0.11 *
45	8.08	280	577	407/289	A-type procyanidin dimer ^b^ VI	n.d.	94.91 ± 0.08 *
47	8.17	280	865	577/289	A-type procyanidin trimer III	n.d.	103.61 ± 0.10 *
49	8.68	280	465	289	(Epi)catechin methyl-hexoside III	n.d.	96.21 ± 1.10 *
50	9.56	280	465	289	(Epi)catechin methyl-hexoside IV	n.d.	28.52 ± 0.07 *
56	11.09	280	893	603/289	(Epi)catechin-ethyl trimer	n.d.	70.45 ± 0.9 *
						Sum 2011.12 ± 9.92	Sum 1795.85 ± 16.78
**Flavonols**							
32	6.39	350	609	301	Quercetin 3-*O*-rutinoside ^b^	92.91 ± 2.05 *	n.d.
35	6.62	340	447	301	Quercetin methyl-pentoside	9.11 ± 0.39 *	n.d.
37	7.13	340	651	446/301	Quercetin acylated-deoxyhexoside I	166.51 ± 1.79 *	n.d.
39	7.18	340	651	447/301	Quercetin acylated-deoxyhexoside II	148.93 ± 1.02 *	n.d.
40	7.22	351	623	315	Isorhamnetin 3-*O*-rutinoside ^b^	459.80 ± 5.61 *	n.d.
42	7.54	355	463	301	Quercetin 3-*O*-galactoside ^b^	59.27 ± 0.24 *	n.d.
44	7.70	355	463	301	Quercetin 3-*O*-glucoside ^b^	98.43 ± 1.88 *	n.d.
46	8.14	340	505	301	Quercetin-acylated-hexoside I	180.03 ± 0.99 *	n.d.
48	8.43	340	505	463/301	Quercetin-acylated-hexoside II	413.14 ± 11.86 *	n.d.
51	9.69	355	463	301	Quercetin 3-*O*-hexoside	n.d.	10.47 ± 0.06 *
52	9.80	353	519	314/299	Isorhamnetin-acylated-hexoside I	4.65 ± 0.12 *	n.d.
53	10.20	350	477	314	Isoramnetin 3-*O*-galactoside ^b^	145.89 ± 2.11 *	n.d.
54	10.27	350	423	287	Kaempferol pentoside	n.d.	6.58 ± 0.09 *
55	10.63	350	519	314/299	Isorhamnetin acylated-hexoside II	32.62 ± 0.88 *	n.d.
57	11.24	353	519	314/299	Isorhamnetin-acylated-hexoside III	234.36 ± 4.29 *	n.d.
58	11.33	346	447	287	Kaempferol 3-*O*-galactoside ^b^	n.d.	12.54 ± 0.07 *
59	11.63	353	519	314/299	Isorhamnetin-acylated-hexoside IV	28.56 ± 1.18 *	n.d.
						Sum2074.21 ± 14.50	Sum29.59 ± 0.13 *
					Sum of phenolic compounds	5575.96 ± 17.80	2330.31 ± 16.98 *

^a^ Experimental data; ^b^ Identified using corresponding authentic standards; ^c^ Values are means ± standard deviation (SD) *n* = 3. Means indicated with an asterisk (*) within lines are different at *p* ≤ 0.05 in Mann–Whitney U-test. Amounts of phenolic acids, flavanols, procyanidins, flavonols and flavanones, were converted into 3-*O*-caffeoylquinic acid (caffeoylquinic acid derivatives), caffeic acid (caffeic acid derivatives), (+)-catechin ((+)-catechin and (−)-epicatechin), epi)catechin methyl-hexoside, (epi)catechin-ethyl trimer), procyanidin B2 (B-type polymeric procyanidins), procyanidin A2 (A-type polymeric procyanidins), quercetin 3-*O*-galactoside (quercetin derivatives), kaempferol 3-*O*-galactoside (kaempferol derivatives), isorhamnetin 3-*O*-glucoside (isorhamnetin derivatives), hesperidin (hesperetin derivatives). Epigallocatechin and quercetin 3-*O*-rutinoside were converted on the basis of the corresponding authentic standards.

**Table 2:** In vitro antioxidant activities in DPPH and ABTS assays of *S. alba* hydromethanolic leaf and bark extracts.

Plant Material	DPPH *	ABTS *
Bark	13.51 ± 0.2 ^a^	21.50 ± 0.32 ^a^
Leaf	28.23 ± 0.6 ^b^	65.41 ± 0.27 ^b^
Ascorbic acid (antioxidant standard)	28.88± 0.21 ^b^	65.43± 0.22 ^b^

The means with the same letter within the columns do not differ significantly according to the Kruskal–Wallis test (*p* ≤ 0.05). The values are means of six replicates ± SD; * EC_50_ the concentration of sample (μg/mL) showing 50% of maximal radical scavenging activity.
